# Effects of Oxygen Manipulation on Myofibroblast Phenotypic Transformation in Patients With Radiation‐Induced Fibrosis

**DOI:** 10.1111/wrr.70075

**Published:** 2025-08-18

**Authors:** Eric V. Mastrolonardo, Sarah Sussman, Bo Yun, Victor Jegede, Dev R. Amin, Joel Rosenbloom, Andrew P. South, Voichita Bar‐Ad, Peter J. Wermuth, Adam J. Luginbuhl

**Affiliations:** ^1^ Department of Otolaryngology–Head and Neck Surgery Thomas Jefferson University Hospital Philadelphia Pennsylvania USA; ^2^ Department of Otolaryngology–Head and Neck Surgery The Ohio State University Wexner Medical Center Columbus Ohio USA; ^3^ Department of Dermatology and Cutaneous Biology The Joan and Joel Rosenbloom Center for Fibrotic Diseases, Thomas Jefferson University Philadelphia Pennsylvania USA; ^4^ Department of Pharmacology, Physiology and Cancer Biology Thomas Jefferson University Philadelphia Pennsylvania USA; ^5^ Department of Radiation Oncology Thomas Jefferson University Hospital Philadelphia Pennsylvania USA

**Keywords:** fibroblast, fibrosis, myofibroblast, oxygen therapy, radiation

## Abstract

We tested if hyperoxic conditions can reduce the proportion of active myofibroblasts, which are assumed to be a major driver of head and neck radiation‐induced fibrosis, as measured by expression levels of pro‐fibrotic genes. Radiated, non‐cancerous soft tissue from the head and neck and skin/soft tissue from non‐radiated flap donor site were collected from each patient. Myofibroblast density was quantified using immunofluorescence staining with α‐SMA and DAPI and visualisation under confocal microscopy and compared between baseline non‐radiated and radiated tissue from the same patient. From each tissue specimen, fibroblast cell lines were cultured and exposed to either normoxic, hypoxic, or hyperoxic conditions for 10 days. Total RNA was extracted and reverse‐transcribed, and gene expression levels were quantified using RT‐PCR. Relative gene expression levels of pro‐fibrotic genes COL1A1, COL3A1, FN‐EDA, α‐SMA, HIF‐1α, VEGFα, and VEGFR were compared between normoxic, hypoxic, and hyperoxic treatment groups. Three patients with six total tissue samples were acquired. Radiated tissue contained a higher density of myofibroblasts (calculated as cells/mm^2^) and demonstrated higher expression of pro‐fibrotic genes than non‐radiated donor site tissue. Hyperoxia decreases expression levels of pro‐fibrotic genes in radiated and non‐radiated tissue, while hypoxia increases pro‐fibrotic gene expression levels in radiated and non‐radiated tissue. Study findings indicate that hypoxia is a driver of myofibroblast activation and that subjects with radiation‐induced fibrosis of the head and neck have increased expression of myofibroblastic phenotype. Hyperoxygenation can reduce the proportion of active myofibroblasts, revealing a potential therapeutic method to halt chronic fibrotic pathways.

## 1 Introduction

Radiation therapy (RT) has remained a cornerstone of treatment for head and neck cancer for decades. However, radiation‐associated toxicity has a profound impact on quality of life and results in an array of symptoms including skin thickening, muscle atrophy, mucosal fibrosis, xerostomia, trismus, and pain. Radiation‐induced fibrosis (RIF) is both a common and significant sequela of RT, particularly in the head and neck. Patients experience a wide range of RIF, and its severity is predictive of long‐term outcomes in overall quality of life, speech, physical function, and even social contact [1, 2]. Recent development and widespread application of intensity‐modulated radiotherapy has served as a critical advancement in reducing side effect burden and severity of RIF. However, RIF remains common amongst a growing population of cancer survivors, and management remains largely limited to supportive care [1, 2, 3].

The mechanism of RIF is similar to chronic wound healing in which an initial injury (ionising radiation) catalyses an inflammatory response, characterised by fibroblast recruitment and overactive extracellular matrix (ECM) deposition. However, prolonged stimulation of these pathways results in excess deposition of collagen and other ECM proteins in a manner that outpaces angiogenesis [4, 5]. The resultant decrease in capillary density exacerbates tissue hypoxia, thus establishing a self‐perpetuating feedback loop [6, 7, 8, 9, 10]. This over‐activation of the inflammatory process is the basis of fibrosis, excessive scarring, and compromised organ function.

We have known for decades that fibrotic tissue change is most directly attributable to the differentiation of quiescent fibroblasts into mature myofibroblasts, over‐producing collagens, fibronectin, and other ECM components [2]. This process, also known as fibroblast activation, or trans‐differentiation, is marked by the appearance of alpha‐smooth muscle actin (α‐SMA), which forms cytoskeletal fibres that confer contractile properties to myofibroblasts. It is this resultant phenotype that causes wound contracture and scarring, which in normal healing is resolved by apoptosis of activated myofibroblasts. It is unclear whether activated myofibroblasts can reacquire a quiescent phenotype. TGF‐β is regarded as the key activator of fibroblast trans‐differentiation and matrix production. In hypoxic tissue, upregulation of TGF‐β has been shown to be induced by HIF‐1α [11]. Unpublished data from our lab has previously demonstrated fibroblasts treated with TGF‐β demonstrate expected myofibroblast activation with increased expression of pro‐fibrotic genes. This serves to validate the proposed mechanism of fibroblast trans‐differentiation and serves as a basis for this study.

Clinically, we observe a range of RIF between patients that appears to worsen over time. To date, we do not have a therapeutic intervention that addresses the underlying pathophysiology and resort to supportive care. There is other research exploring mechanisms to inform the radiated fibroblasts that the repair is complete, signalling either apoptosis or a return to a resting state with a reduced risk forlong‐term RIF syndrome. The focus of this research highlights the phenotypic transformation of the dermal fibroblast as the primary culprit in this debilitating consequence, RIF. We demonstrate that hypoxia is a critical factor inducing myofibroblast activation via TGF‐β and HIF‐1α and that hyperoxygenation reduces the proportion of active myofibroblasts, thus halting the chronic fibrotic pathways in previously radiated tissue derived from patients.

## 2 Materials and Methods

### 2.1 Patient Population

After obtaining approval from the Thomas Jefferson Sidney Kimmel Cancer Center Institutional Review Board (IRB), we collected tissue samples from eligible patients at Thomas Jefferson University Hospital from December 2022 to May 2023. Written consents were obtained. Eligible patients had a history of previous radiation treatment to the head and neck at least 3 months prior to enrollment and were scheduled for post‐radiation surgery with reconstruction planned utilising non‐radiated tissue.

We collected 2 tissue samples from each patient: non‐cancerous, radiated soft tissue from the head and neck (Radiated) and non‐radiated flap donor site soft tissue (Non‐radiated). During our study period, we consented 6 patients and collected 2 samples from each patient, resulting in an initial total of 12 tissue samples. Of these collected specimens, samples from 3 patients had successful culture of fibroblasts, based on abundance of cell growth, and therefore yielded a total of 6 tissue samples for analysis. Patient characteristics, surgical, and medical history surrounding previous radiation treatment and salvage surgery were prospectively collected including site of radiation, non‐radiated donor site, time since previous radiation therapy, type of radiation, and radiation dose.

### 2.2 Intraoperative Tissue Sample Collection and Fibroblast Culture Creation

Tissue samples were transferred to the laboratory within 30 min of harvest. Samples were divided, stored in liquid nitrogen for total RNA isolation, frozen in Optimal Cutting Temperature Compound (Electron Microscopy Services, Hatfield, PA), and processed for immunohistochemistry. The remaining piece was processed for cell culture by incubation in media consisting of DMEM, fetal bovine serum, penicillin/streptomycin, ascorbic acid 2‐phosphate, and collagenase II (Worthington). Isolated cells were sub‐cultured and obtained for both analyses and storage. Cells were pelleted by centrifugation, resuspended in the DMEM media, and plated in a culture dish coated with a 3 kPa polydimethylsiloxane (PDMS) hydrogel. We isolated and maintained cells in culture on the soft hydrogel matrix with stiffness of 3 kPa to approximate the stiffness of normal skin.

### 2.3 Experimental Oxygen Levels and Cell Cultures

We used 6 primary cell populations (Non‐Radiated and Radiated from 3 patients), each with 3 experimental replicates. Each cell population was cultured and grown within the BioSpherix Chamber set at 37°C under either normoxia (21% O_2_, 5% CO_2_, 74% N_2_), hypoxia (1% O_2_, 5% CO_2_, 94% N_2_), or hyperoxia (80% O_2_, 5% CO_2_, 15% N_2_) for 10 days (C‐Chamber Incubator Subchamber, BioSpherix Ltd).

### 2.4 Reverse Transcription Polymerase Chain Reaction (RT‐PCR)

Total RNA was then isolated employing the Trizol Reagent (Invitrogen, Waltham, MA) and reverse‐transcribed using the Invitrogen SuperScript IV First‐Strand Synthesis System protocol (Thermo Fisher Scientific Inc.). Total RNA concentration was determined by UV spectroscopy at 260/280 nm. Total RNA (250 ng) was annealed to 50 ng/μL hexamers in the presence of 10 mM dNTP mix. A reaction mix was added to each tube, and the samples were incubated at 23°C followed by 55°C. The resulting cDNA was diluted for use in RT‐PCR analysis to assess pro‐fibrotic genes expression (PFGE) levels. The pro‐fibrotic gene panel consisted of the following: *collagen 1, subunit alpha 1* (*COL1A1*)*; collagen 3, subunit alpha 1* (*COL3A1*)*; alpha smooth muscle actin* (*α‐SMA*)*; fibronectin‐EDA* (*FN‐EDA*)*; transforming growth factor‐beta* (*TGF‐β*)*; hypoxia inducible factor‐1 alpha* (*HIF‐1α*)*; vascular endothelial growth factor alpha* (*VEGFα*); and *VEGF receptor* (*VEGFR*).

Gene expression was measured for triplicate wells for each treatment condition for each cell line, and RNA from each well was measured in triplicate. A 2 μL aliquot of cDNA was used in each reaction along with 200 nanomoles of forward and reverse gene‐specific primers for PCR analysis (Thermo Fisher QuantStudio 12 K Flex Real‐Time PCR System). Samples underwent 40 amplification cycles at 95°C followed by 1 min at 60°C. This was followed by melt curve analysis to verify primer specificity.

The cycle threshold (Ct) value was used to quantify the expression level of each gene. The 18S gene was used as the housekeeping gene, and the assumption was made that the value of 18S RNA in a sample reflected the amount of total RNA in a sample. We calculated relative Ct values to compare the expression level of each gene relative to 18S between different oxygenation conditions. The expression level of each gene from Non‐Radiated cells cultured under normoxic conditions was arbitrarily set as the standard at 100% for comparison; the amount of gene expression from other samples was calculated relative to these levels.

### 2.5 Myofibroblast Density

Tissue sections (Radiated and Non‐Radiated) from each patient that were designated for myofibroblast density analysis were frozen with optical cutting temperature compound and sectioned using a cryostat maintained at‐24°C. The sections were first co‐stained with primary antibodies (anti‐α‐SMA and anti‐fibronectin) and co‐stained again with secondary antibodies: 488 nm goat anti‐rabbit (for fibronectin) and 594 nm goat anti‐mouse (for α‐SMA). The sections were then incubated at room temperature. Tissues were visualised using immunofluorescence confocal microscopy (Nikon A1R) and NIS‐Elements program. The number of α‐SMA+ cells/unit area for each sample was quantified using NIH FIJI software to count the number of myofibroblasts and calculate myofibroblast density [number of myofibroblasts/unit area].

### 2.6 Statistical Analysis

Standard summary statistics (mean, standard deviation, standard error, confidence intervals) were used to summarise variables as appropriate. Baseline Ct values were compared between Radiated versus Non‐radiated tissue and between different oxygenation conditions (hyperoxia versus normoxia, hypoxia versus normoxia, and hyperoxia versus hypoxia). Paired t‐test analysis was used to compare differences in each gene expression level per different oxygen treatments. Hypothesis tests were 2‐sided. All statistical analyses were completed using IBM SPSS Statistics 28.0.1.1 (IBM, Chicago, IL). Statistical significance was defined as *p* < 0.05.

## 3 Results

### 3.1 Sample Characteristics

Baseline clinical characteristics of tissue samples are described in Table 1. Radiated sites included the oral cavity and oropharynx. Non‐radiated flap donor sites included the skin and soft tissue superficial to the scapula and the thigh. Fibrosis grades ranged from no clinical evidence of fibrosis to Grade 4 fibrosis (according to CTCAE version 5.0). Samples were collected 16–57 months since completion of radiation treatment.

**TABLE 1:** Baseline characteristics.

Subject	Radiated site	Non‐radiated flap donor site (soft tissue)	Fibrosis grade (CTCAE v 5.0)	Time since radiation (Months)
1	Oral Cavity	Scapula	2	31
2	Oropharynx	Thigh	n/a*	16
3	Oral Cavity	Scapula	4	57

* No clinical evidence of fibrosis.

### 3.2 Baseline Myofibroblast Density Analysis

**FIGURE 1:**
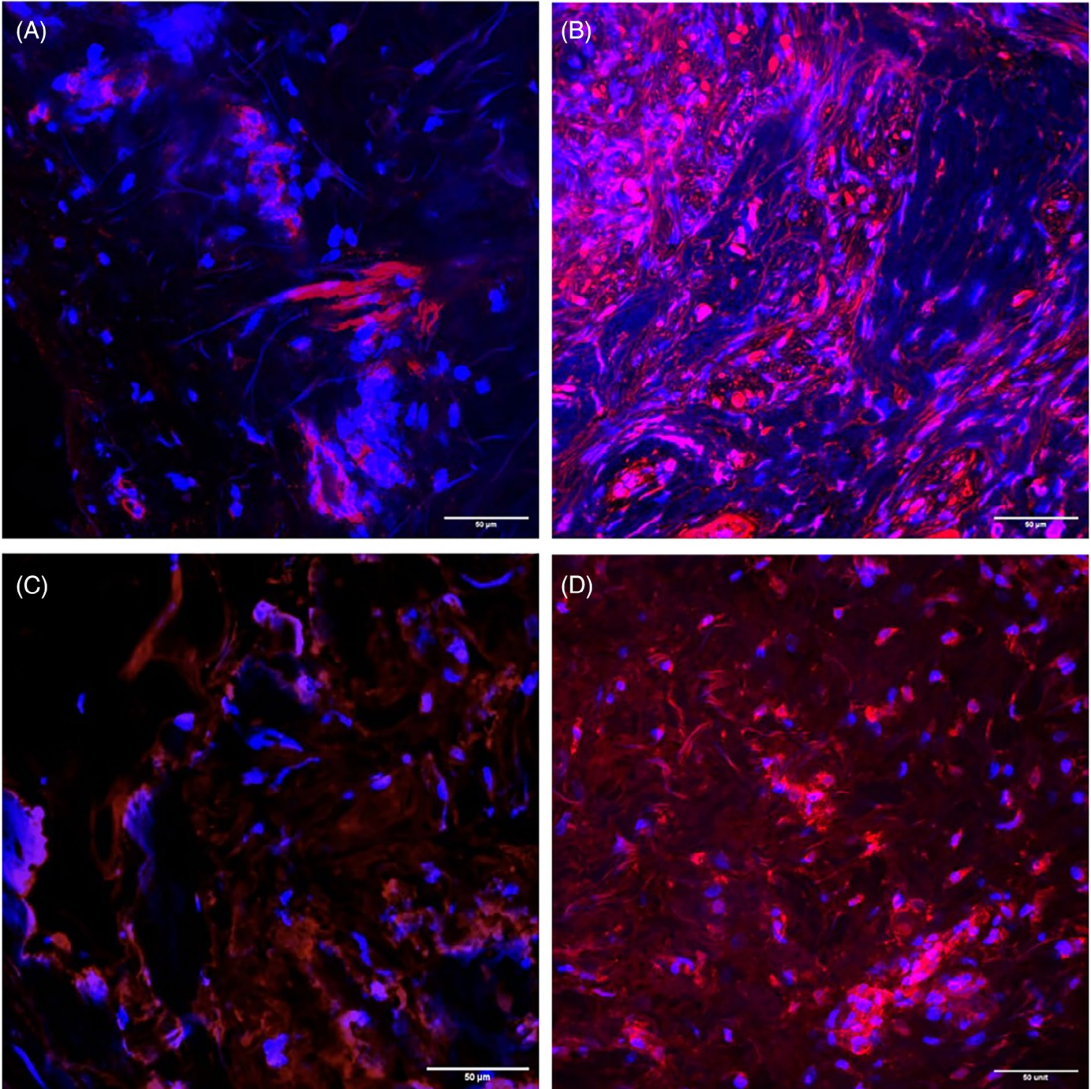
α‐SMA and DAPI Stains for Myofibroblast Density Comparison. Immunofluorescence images were acquired with confocal microscope at 40× magnification. Nuclei were stained with DAPI (blue channel), and myofibroblasts were stained with α‐SMA (red channel). Scale bar, 50 μm. Areas of concentrated expression α‐SMA were considered to be blood vessels and were not counted as nuclei for myofibroblast density analysis. (A) Subject 1 Non‐radiated tissue versus (B) Subject 1 Radiated tissue. (C) Subject 3 Non‐radiated tissue versus (D) Subject 3 Radiated tissue.

To better understand tissue composition and the myofibroblast distribution of each tissue at baseline, a piece of each specimen was sampled and analysed using immunohistochemistry (immunofluorescence confocal microscopy) prior to the introduction of any oxygen intervention. The number and density of myofibroblasts per unit area are described in Table 2 and visualised in Figure 1. In tissue samples from Subjects 1 and 3, radiated tissue contained a notably higher density of myofibroblasts (calculated as cells/mm^2^). Tissue from Subject 2 could not be analysed due to the mounted tissue sample being too small to visualise a sufficient number of nuclei under the microscope for analysis.

**TABLE 2:** Myofibroblast density counts.

Sample	Myofibroblast density (cells/mm^2^)	Myofibroblast nuclei/all nuclei
Subject 1 non‐radiated tissue	2 cells/mm^2^	12/40 = 0.3
Subject 1 radiated tissue	93 cells/mm^2^	558/602 = 0.93
Subject 3 non‐radiated tissue	2.5 cells/mm^2^	15/39 = 0.38

### 3.3 Radiated Tissue Demonstrates Higher Expression of Pro‐Fibrotic Genes

**FIGURE 2:**
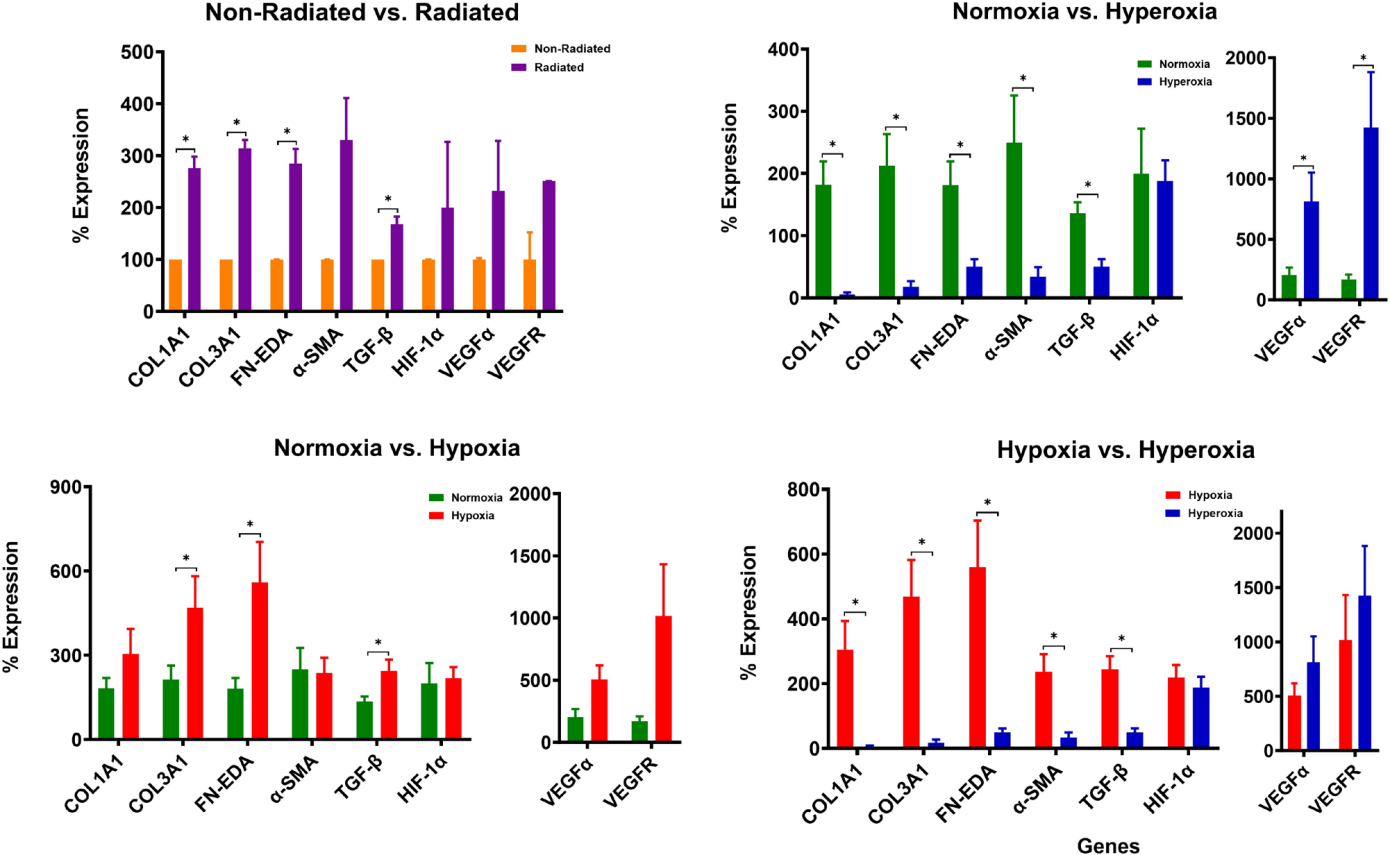
Pro‐fibrotic and HIF‐responsive gene expression levels. (A) Non‐Radiated versus Radiated. Baseline gene expression levels comparing Non‐radiated (orange) versus Radiated (purple) tissue. (B–D) Gene expression levels from both Non‐radiated and Radiated tissue samples in combined analysis under (B) Normoxia versus Hyperoxia: Normoxic conditions (green) versus hyperoxic conditions (blue), (C) Normoxia versus Hypoxia: Normoxic conditions (green) versus hypoxic conditions (red), and (D) Hypoxia versus Hyperoxia: Hyperoxic conditions (blue) versus hypoxic conditions (red).

COL1A1 (*p* = 0.019), COL3A1 (*p* = 0.005), FN‐EDA (*p* = 0.029), and TGF‐β (*p* = 0.039) demonstrated significantly higher expression in Radiated tissue than Non‐radiated tissue at baseline (Figure 2A). α‐SMA demonstrated a trend in increasing gene expression in Radiated tissue compared to Non‐Radiated tissue but did not achieve statistical significance (*p* = 0.066) (Figure 2A). HIF‐1α, VEGFα, and VEGFR demonstrated similar expression levels in Radiated and Non‐Radiated tissue at baseline.

### 3.4 Hyperoxic Conditions Lead to a Decrease in Expression of Pro‐Fibrotic Genes Relative to Normoxic Conditions

Hyperoxic conditions decreased expression levels of COL1A1 (*p* = 0.007), COL3A1 (*p* = 0.017), FN‐EDA (*p* = 0.045), α‐SMA (*p* = 0.047), and TGF‐β (*p* < 0.001) compared to normoxic conditions in both Radiated and Non‐Radiated tissue (Figure 2B). HIF‐1α demonstrated similar expression levels in hyperoxic and normoxic conditions. Hyperoxic conditions increased expression levels of VEGFα (*p* = 0.028) and VEGFR (*p* = 0.037) (Figure 2B). Effects of hyperoxia on pro‐fibrotic, HIF‐1α, and HIF‐1α‐responsive gene expression in either Non‐Radiated versus Radiated tissues are separately described in (S1 and Figure 1). No differences in cell numbers were observed between normoxic and hypoxic culture conditions.

### 3.5 Hypoxic Conditions Lead to an Increase in Expression of Pro‐Fibrotic Genes Relative to Normoxic Conditions

Hypoxia increased expression levels of COL3A1 (*p* = 0.013), FN‐EDA (*p* = 0.019), and TGF‐β (*p* = 0.027) compared to normoxic conditions in both tissue Radiated and Non‐Radiated tissue (Figure 2C). Both COL1A1 (*p* = 0.085) and α‐SMA (*p* = 0.763) demonstrated similar expression levels in hypoxic and normoxic conditions. HIF‐1α, VEGFα, and VEGFR demonstrated similar expression levels in hypoxic and normoxic conditions (Figure 2C). Effects of hypoxia on pro‐fibrotic, HIF‐1α, and HIF‐1α‐responsive gene expression in either Radiated or Non‐radiated tissue are separately described (S2 and Figure 2). No differences in cell numbers were observed between normoxic and hyperoxic culture conditions.

### 3.6 Hyperoxic Conditions Lead to a Decrease in Expression of Pro‐Fibrotic Genes Relative to Hypoxic Conditions

Hyperoxia decreased expression levels of COL1A1 (*p* = 0.021), COL3A1 (*p* = 0.011), FN‐EDA (*p* = 0.022), α‐SMA (*p* = 0.014), and TGF‐β (*p* = 0.005) compared to hypoxic conditions (Figure 2D). HIF‐1α, VEGFα, and VEGFR demonstrated statistically similar expression levels in hypoxic and hyperoxic conditions (Figure 2D). Compared to hypoxic conditions, the effects of hyperoxia on pro‐fibrotic, HIF‐1α, and HIF‐1α‐responsive gene expression in either radiated or Non‐radiated tissue are separately described (Figure S2 and Figure S3).

## 4 Discussion

We sought to induce differentiation of quiescent fibroblasts into active myofibroblasts by modulating oxygen concentrations and measuring subsequent changes in pro‐fibrotic gene expression. At baseline, we found that radiated tissue exhibits higher levels of pro‐fibrotic gene expression and consists of a higher density of myofibroblasts compared to non‐radiated tissue. In addition, hyperoxic conditions down‐regulated expression of pro‐fibrotic genes, thus indicating a reduction in the proportion of active myofibroblasts, while the converse was true of hypoxic conditions. Effects of radiation and hyperoxia/hypoxia on pro‐fibrotic gene expression are summarised in Table 3.

**TABLE 3:** Summary of gene expression levels.

	Baseline	Hypoxia	Hyperoxia	Difference in mean (radiated—Non‐radiated)	Difference in average expression level (radiated—non‐radiated)
COL1A1	—	+123.4 ↑	−176.1 ↓*	163.2*	↑*
COL3A1	—	+256.4 ↑*	−176.1 ↓*	224.8*	↑*
FN‐EDA	—	+378.1↑*	−130.9 ↓*	162.2*	↑*
HIF1α	—	+18.6 ↑	−11.92 ↓	199.3	↑
α‐SMA	—	−12.8 ↓	−215.5 ↓*	298.9*	↑*
TGF*β*	—	+107.8 ↑*	−86.2 ↓*	72.5*	↑*
VEGF	—	+300.6 ↑	+606.3 ↑	203.3	↑
VEGFR	—	+845.6 ↑	+1253.1↑	203.3	↑

*Note:* Difference was calculated as the mean gene expression level seen based on Radiation and Oxygenation statuses.

* Indicates trends with *p* < 0.05.

Ionising radiation induces vascular injury, which is a key component in the pathogenesis of RIF. This phenomenon is the foundation of our work. In short, endothelial cells are particularly sensitive to radiation, and the sub‐endothelial ECM components are exposed to platelets that promote an antifibrinolytic‐coagulation cascade that triggers clotting and ultimately results in vascular occlusion [12]. Subsequent damage to the vascular endothelial barrier increases vascular permeability, thereby stimulating the release of chemokines including VEGF, platelet‐derived growth factor (PDGF), and basic fibroblast growth factor (bFGF) which promote proliferation and recruitment of inflammatory cells. This process catalyses the conversion of fibroblasts to become myofibroblasts [13, 14]. Excessive collagen deposition leading to luminal stenosis can further exacerbate tissue ischemia and hypoxia, which may stimulate free radical production [15]. HIF‐1a induces the expression of profibrotic mediators—endothelin‐1 (ET‐1), VEGF, CTGF, bFGF, and TGF‐β1 [16]. While VEGF ordinarily promotes neoangiogenesis, chronic hypoxia and excess deposition of collagen decrease vascularity. This makes fibrotic tissue even more vulnerable to trauma and ischemia, instigating a positive feedback loop of further myofibroblast activation and collagen deposition, eventually leading to loss of function, tissue atrophy, or necrosis [17].

The myofibroblast has been the center of extensive research into the dysregulated chronic inflammatory cycle and development of RIF, as well as the implication of hypoxia in the pathogenesis of RIF. In our study, at baseline normoxic conditions, the radiated tissue expressed higher levels of COL1A1, COL3A1, FN‐EDA, and TGF‐β compared to Non‐Radiated donor tissue. Furthermore, immunofluorescence confocal microscopy and direct visualisation of myofibroblasts revealed radiated specimens contained an increased number of myofibroblasts per mm^2^ compared to their associated Non‐Radiated donor tissue counterparts (Figure 1). These findings are consistent with previous research describing myofibroblast activation in response to tissue injury with the intent to repair damaged ECM. In normal wound healing, myofibroblasts are deactivated or cleared by apoptosis once tissue repair is complete; however, dysregulation of this process by chronic stress secondary to RT can cause persistent myofibroblast activation, which subsequently leads to excessive and disorganised ECM repair, collagen accumulation, contraction, and eventual fibrosis [18, 19, 20, 21].

The complex nature of cellular interactions in these populations must also be considered. Though outside of the scope of this study, myofibroblasts' inter‐ and intracellular interactions (e.g., with pericytes and/or endothelial cells) also likely play a role in the development of fibrosis. Interestingly, pericytes are believed to differentiate into vascular smooth muscle cells and vice versa and may even give rise to other types of mesenchymal cells, including fibroblasts [22]. Furthermore, pericyte–myofibroblast transition has been linked to promoting subretinal fibrosis, renal fibrosis, and pulmonary fibrosis [23, 24]. Although we did not investigate the role of pericytes on myofibroblasts and pro‐fibrotic gene expression, we could postulate how such interactions can affect the formation of fibrosis and beyond.

As EMT is at the cornerstone of the pathogenesis of fibrosis, studies proving HIF‐1α to stimulate EMT in both in vitro and in vivo models are fundamental to our work and present study [25, 26, 27, 28]. In a renal model, Higgens et al. found correlations between HIF‐1α expression and the degree of fibrosis in renal biopsy tissues from patients with chronic kidney disease through increased expression of ECM‐modifying and lysyl oxidase genes (specifically Lox and LoxL2) and by enhancing the transition of epithelial cells toward a mesenchymal phenotype. Similar to our findings, epithelial cells cultured under hypoxic conditions (1% O2) exhibited increased expression of α‐SMA and their morphology was consistent with epithelial cells undergoing EMT to produce a more fibroblast‐like cell type. They concluded that HIF‐1α‐mediated expression of lysyl oxidases in hypoxic tubular epithelial cells enables the transition toward an activated fibroblast‐like phenotype, migration toward the interstitium, and promotion of fibrosis [28].

Recent evidence suggests that the myofibroblast phenotype can be reversed. Studies have demonstrated that this loss of myofibroblast phenotype can be induced with various TGF‐β inhibitors in vitro and in animal models in vivo [29]. However, there has been universal difficulty applying these agents as potential therapies in vivo given the pleiotropic activities of TGF‐β in normal tissue repair. HIF‐1α signalling can trigger pro‐fibrotic gene expression, rendering it a potential target in other diseases mediated by chronic fibrosis, including diabetic nephropathy and idiopathic lung fibrosis [30, 31]. Further, HIF‐1α may be readily suppressed by existing therapies such as hyperbaric oxygen therapy and pharmaceuticals that modify cellular oxygen consumption.

The focus of this research highlights the phenotypic transformation of myofibroblasts through manipulation of oxygen levels. Our results suggest that hypoxic conditions drive fibroblast trans‐differentiation into myofibroblasts and that hyperoxic conditions reduce the proportion of active myofibroblasts, since no observable cell death was observed to explain decreased PFGE levels. This effect is likely mediated by the effect of hyperoxia on TGF‐β [4, 11]. These findings suggest that hyperoxia plays a critical role in de‐differentiating myofibroblasts and thus may halt the chronic, feedforward cascade of pro‐fibrotic pathways that are triggered by factors like TGF‐β and HIF‐1α [32, 33]. As anticipated, hypoxia increased expression levels of COL3A1, FN‐EDA, and TGF‐β compared to normoxia conditions but did not significantly increase levels in COL1A1 and α‐SMA. While the lack of significantly increased COL1A1 and α‐SMA gene expression warrants further investigation, these findings overall suggest that hypoxia does in fact trigger fibroblast trans‐differentiation into active myofibroblasts. While the pro‐fibrotic genes are more reliable to measure, the HIF‐responsive genes are not as clearly defined. When analysing HIF‐1α and HIF‐1α‐target genes (VEGFα and VEGFR), hypoxia resulted in a statistically similar *upregulation* in expression for all 3 genes while hyperoxia also saw a statistically similar upregulation in expression for VEGFα and VEGFR but a statistically insignificant decrease in HIF‐1α (Figure 2B,C). Furthermore, despite the decrease in expression levels of other pro‐fibrotic genes, the expression levels of HIF‐1α were unchanged when comparing between Non‐radiated versus Radiated tissues (Figure S3). While hypoxia is a well‐studied inducer of HIF‐1α, these findings support the view that HIF‐1α is not strictly a hypoxia‐response gene but instead a general sensor of oxygen levels whose expression is triggered by changes in oxygen concentration. To examine this phenomenon, Terraneo et al. tested if hyperoxia and hypoxia have divergent effects on HIF‐1α expression in vivo in mice xenografts [34]. They discovered that hyperoxia paradoxically doubled HIF‐1α expression compared to normoxia and hypoxia, likely secondary to the marked cell response to redox imbalance; this was evidenced by the associated increase in nuclear factor (erythroid‐derived 2)‐like 2 (Nrf2), the well‐described upstream activator of HIF‐1α [35]. In their study, hyperoxia also led to overexpression of VEGFα and VEGFR [34, 35]. While further investigation of hyperoxia's effects on HIF‐1α's upstream activators and inhibitors was not conducted within this set of experiments, these results demonstrate the need for additional hyperoxic investigation.

Although these experiments demonstrate that hypoxia, relative to normoxia, increased HIF‐1α expression in Non‐Radiated donor tissue, this increase did not achieve statistical significance (Table 3). When exposed to hypoxic conditions, we observed that Radiated tissues actually demonstrated a slight decrease in HIF‐1α compared to their Non‐radiated tissue counterparts. This may suggest that the prolonged hypoxic state in Radiated tissues may limit the effects of additional hypoxia on HIF‐1α levels seen in our experiments. A recent study by Jaśkiewicz observed destabilisation of HIF‐1α mRNA during prolonged hypoxia of 24 h due to re‐stabilisation of prolyl hydroxylase activity [36]. Although the conditions in this previous study do not parallel the hypoxic environment created in In vivo radiated tissues, the results support the hypothesis that radiation and subsequent hypoxic changes may have influenced the observed changes in HIF‐1α levels.

This study has several limitations to address. A main limitation of this study is our small sample size due to a small number of patients selected as well as inherent limitations of culturing and plating fibroblast which may be slow to reach confluence. We attempted to address this via two methods. First, we sampled non‐radiated donor tissue from each subject to serve as a control when performing paired statistical analysis to mitigate person‐to‐person variability. Second, we utilised a total of 3 experimental replicates with an associated 3 technical replicates, resulting in 9 data points for each cell line and oxygen conditions. Looking forward, we look to increase our n and further stratify specimens by clinical fibrosis grading, total radiation dose, radiation site, and elapsed time since last radiation dose to better understand how these factors also affect degrees of radiation fibrosis and myofibroblast density and activity.

As previously mentioned, our data demonstrate that hyperoxia reduces the proportion of active myofibroblasts in vitro; future study is warranted to investigate if this change is driven by myofibroblast de‐differentiation back to the quiescent fibroblast phenotype or via another mechanism. There is literature to suggest phenotypic reversion is possible, but this research has yet to be completed on radiated tissue [37, 38]. Our future experimental model would require baseline measurements that would define the quantitative and qualitative presence of myofibroblasts followed by tracking their behaviour through the therapeutic conditions. Another limitation of the present study is related to the timing and duration of experimental conditions. While we hypothesise that the observed in vitro changes in gene expression will be similar in vivo, this requires confirmation. The timing of hypoxic and hyperoxic conditions is based on feasibility in the laboratory. These data reflect the cell state at a single time point. Longer periods under hypoxic conditions might better model chronic effects of hypoxia in vivo; however, owing to time and feasibility of maintaining cell cultures for long time periods, we selected a 10‐day period. Lastly, we are unable to manipulate barometric pressure in the BioSpherix chamber, thus the hyperoxic conditions may not be directly comparable to the effects of hyperbaric oxygen therapy in patients.

## 5 Conclusion

Our findings confirm that hypoxia is a driver of myofibroblast activation and subsequent collagen production, and that subjects with RIF have increased expression of the myofibroblastic phenotype. We have demonstrated that fibroblasts placed in hypoxic conditions will become activated myofibroblasts and that hyperoxygenation reduces the proportion of active myofibroblasts, thus potentially revealing a therapeutic target to halt the chronic fibrotic pathways.

## Conflicts of Interest

The authors declare no conflicts of interest.

## Supporting information


**Figure S1:** Profibrotic and HIF‐responsive gene expression levels in Normoxia versus Hyperoxia. (A) Non‐radiated: Normoxia versus Hyperoxia. Gene expression levels under normoxic conditions (green) versus hyperoxic conditions (blue) in Non‐radiated tissue. (B) Radiated tissue: Normoxia versus Hyperoxia. Gene expression levels under normoxic conditions (green) versus hyperoxic conditions (blue) in Radiated tissue.

**Figure S2:** Profibrotic and HIF‐responsive gene expression levels in Normoxia versus Hypoxia. (A) Non‐Radiated Tissue: Normoxia versus Hypoxia. Gene expression levels under normoxic conditions (green) versus hypoxic conditions (red) in Non‐radiated tissue. (B) Radiated Tissue: Normoxia versus Hypoxia: Gene expression levels under normoxic conditions (green) versus hypoxic conditions (red) in Radiated tissue.

**Figure S3:** Profibrotic and HIF‐responsive gene expression levels in Hypoxia versus Hyperoxia. (A) Non‐Radiated Tissue: Hypoxia versus Hyperoxia. Gene expression levels under hypoxic conditions (red) versus hyperoxic conditions (blue) in Non‐Radiated tissue and (B) Radiated Tissue: Hypoxia versus Hyperoxia. Gene expression levels under hypoxic conditions (red) versus hyperoxic conditions (blue) in Radiated tissue.

## Data Availability

The data that support the findings of this study are available from the corresponding author upon reasonable request.

## References

[1] P. Ramia , L. Bodgi , D. Mahmoud , et al., “Radiation‐Induced Fibrosis in Patients With Head and Neck Cancer: A Review of Pathogenesis and Clinical Outcomes,” Clinical Medicine Insights. Oncology 16 (2022): 11795549211036898. 35125900 10.1177/11795549211036898 PMC8808018

[2] M. Martin , J. L. Lefaix , and S. Delanian , “TGF‐β1 and Radiation Fibrosis: A Master Switch and a Specific Therapeutic Target?,” International Journal of Radiation Oncology, Biology, Physics 47, no. 2 (2000): 277–290. 10802350 10.1016/s0360-3016(00)00435-1

[3] C. B. V. de Andrade , I. P. R. Ramos , A. C. N. de Moraes , et al., “Radiotherapy‐Induced Skin Reactions Induce Fibrosis Mediated by TGF‐β1 Cytokine,” Dose Response [Internet] 15, no. 2 (2017): 1559325817705019. 28507463 10.1177/1559325817705019 PMC5415163

[4] I. A. Darby and T. D. Hewitson , “Hypoxia in Tissue Repair and Fibrosis,” Cell and Tissue Research 365, no. 3 (2016): 553–562. 27423661 10.1007/s00441-016-2461-3

[5] K. Richter and T. Kietzmann , “Reactive Oxygen Species and Fibrosis: Further Evidence of a Significant Liaison,” Cell and Tissue Research 365, no. 3 (2016): 591–605. 27345301 10.1007/s00441-016-2445-3 PMC5010605

[6] J. M. Straub , J. New , C. D. Hamilton , C. Lominska , Y. Shnayder , and S. M. Thomas , “Radiation‐Induced Fibrosis: Mechanisms and Implications for Therapy,” Journal of Cancer Research and Clinical Oncology 141, no. 11 (2015): 1985–1994. 25910988 10.1007/s00432-015-1974-6 PMC4573901

[7] B. D. Kelly , S. F. Hackett , K. Hirota , et al., “Cell Type–Specific Regulation of Angiogenic Growth Factor Gene Expression and Induction of Angiogenesis in Nonischemic Tissue by a Constitutively Active Form of Hypoxia‐Inducible Factor 1,” Circulation Research 93, no. 11 (2003): 1074–1081. 14576200 10.1161/01.RES.0000102937.50486.1B

[8] B. Wang , J. Wei , L. Meng , et al., “Advances in Pathogenic Mechanisms and Management of Radiation‐Induced Fibrosis,” Biomedicine & Pharmacotherapy 121 (2020): 109560. 31739160 10.1016/j.biopha.2019.109560

[9] H. Burger , M. Loffler , and H. P. A. Bamberg , “Molecular and Cellular Basis of Radiation Fibrosis,” International Journal of Radiation Biology 73, no. 4 (1998): 401–408. 9587078 10.1080/095530098142239

[10] S. Delanian , M. Martin , A. Bravard , C. Luccioni , and J. L. Lefaix , “Abnormal Phenotype of Cultured Fibroblasts in Human Skin With Chronic Radiotherapy Damage,” Radiotherapy and Oncology 47, no. 3 (1998): 255–261. 9681888 10.1016/s0167-8140(97)00195-3

[11] J. Yarnold and M. C. Vozenin Brotons , “Pathogenetic Mechanisms in Radiation Fibrosis,” Radiotherapy and Oncology 97, no. 1 (2010): 149–161. 20888056 10.1016/j.radonc.2010.09.002

[12] A. I. Soloviev , S. M. Tishkin , A. V. Parshikov , I. V. Ivanova , E. V. Goncharov , and A. M. Gurney , “Mechanisms of Endothelial Dysfunction After Ionized Radiation: Selective Impairment of the Nitric Oxide Component of Endothelium‐Dependent Vasodilation,” British Journal of Pharmacology 138, no. 5 (2003): 837–844. 12642385 10.1038/sj.bjp.0705079 PMC1573711

[13] D. Abraham and O. Distler , “How Does Endothelial Cell Injury Start? The Role of Endothelin in Systemic Sclerosis,” Arthritis Research & Therapy 9, no. Suppl 2 (2007): S2. 10.1186/ar2186 PMC2072886 17767740

[14] M. M. Kopaniak , A. C. Issekutz , and H. Z. Movat , “Kinetics of Acute Inflammation Induced by E Coli in Rabbits. Quantitation of Blood Flow, Enhanced Vascular Permeability, Hemorrhage, and Leukocyte Accumulation,” American Journal of Pathology 98, no. 2 (1980): 485–498. 6986785 PMC1903407

[15] R. Rathore , Y. Zheng , C. Niu , et al., “Hypoxia Activates NADPH Oxidase to Increase [ROS]i and [Ca2+]i Through the Mitochondrial ROS‐PKCɛ Signaling Axis in Pulmonary Artery Smooth Muscle Cells,” Free Radical Biology & Medicine 45 (2008): 1223–1231. 18638544 10.1016/j.freeradbiomed.2008.06.012 PMC2586914

[16] M. Sharma and R. Radhakrishnan , “CTGF Is Obligatory for TGF‐β1 Mediated Fibrosis in OSMF,” Oral Oncology 56 (2016): e10–e11. 27036370 10.1016/j.oraloncology.2016.03.011

[17] Y. Taha , Y. Raab , A. Larsson , et al., “Vascular Endothelial Growth Factor (VEGF)—A Possible Mediator of Inflammation and Mucosal Permeability in Patients With Collagenous Colitis,” Digestive Diseases and Sciences 49, no. 1 (2004): 109–115. 14992444 10.1023/b:ddas.0000011611.92440.f2

[18] L. Hecker , R. Jagirdar , T. Jin , and V. J. Thannickal , “Reversible Differentiation of Myofibroblasts by MyoD,” Experimental Cell Research 317, no. 13 (2011): 1914–1921. 21440539 10.1016/j.yexcr.2011.03.016 PMC3123424

[19] B. Hinz , “Formation and Function of the Myofibroblast During Tissue Repair,” Journal of Investigative Dermatology 127, no. 3 (2007): 526–537. 17299435 10.1038/sj.jid.5700613

[20] A. Desmoulire , M. Redard , I. Darby , and G. Gabbiani , “Apoptosis Mediates the Decrease in Cellularity During the Transition Between Granulation Tissue and Scar,” American Journal of pathology 146 (1995): 56–66. 7856739 PMC1870783

[21] S. E. Wilson , S. S. Chaurasia , and F. W. Medeiros , “Apoptosis in the Initiation, Modulation and Termination of the Corneal Wound Healing Response,” Experimental Eye Research 85, no. 3 (2007): 305–311. 17655845 10.1016/j.exer.2007.06.009 PMC2039895

[22] A. Armulik , A. Abramsson , and C. Betsholtz , “Endothelial/Pericyte Interactions,” Circulation Research 97, no. 6 (2005): 512–523. 16166562 10.1161/01.RES.0000182903.16652.d7

[23] C. Hung , G. Linn , Y.‐H. Chow , et al., “Pericyte‐Myofibroblast Transition in the Human Lung,” Biochemical and Biophysical Research Communications 528, no. 2 (2020): 379–385. 10.1016/j.bbrc.2020.05.091 32473754

[24] Y. Li , Y. Song , L. Zhao , et al., “Novel Mechanism of the Pericyte‐Myofibroblast Transition in Renal Interstitial Fibrosis: Core Fucosylation Regulation,” Scientific Reports 7 (2017): 16914. 29209018 10.1038/s41598-017-17193-5 PMC5717002

[25] M. Iwano , D. Plieth , T. M. Danoff , C. Xue , H. Okada , and E. G. Neilson , “Evidence That Fibroblasts Derive From Epithelium During Tissue Fibrosis,” Journal of Clinical Investigation 110, no. 3 (2002): 341–350. 12163453 10.1172/JCI15518 PMC151091

[26] M. Iwano and E. G. Neilson , “Mechanisms of Tubulointerstitial Fibrosis,” Current Opinion in Nephrology and Hypertension 13, no. 3 (2004): 279–284. 15073485 10.1097/00041552-200405000-00003

[27] R. Kalluri and E. G. Neilson , “Epithelial‐Mesenchymal Transition and Its Implications for Fibrosis,” Journal of Clinical Investigation 112, no. 12 (2003): 1776–1784. 14679171 10.1172/JCI20530 PMC297008

[28] D. F. Higgins , K. Kimura , W. M. Bernhardt , et al., “Hypoxia Promotes Fibrogenesis In Vivo via HIF‐1 Stimulation of Epithelial‐To‐Mesenchymal Transition,” Journal of Clinical Investigation 117 (2007): 3810–3820. 18037992 10.1172/JCI30487 PMC2082142

[29] R. B. Nahomi and R. H. Nagaraj , “The Role of HIF‐1α in the TGF‐β2‐Mediated Epithelial‐To‐Mesenchymal Transition of Human Lens Epithelial Cells,” Journal of Cellular Biochemistry 119, no. 8 (2018): 6814–6827. 29693273 10.1002/jcb.26877 PMC6605039

[30] R. Bessho , Y. Takiyama , T. Takiyama , et al., “Hypoxia‐Inducible Factor‐1α Is the Therapeutic Target of the SGLT2 Inhibitor for Diabetic Nephropathy,” Scientific Reports 9, no. 1 (2019): 14754. 31611596 10.1038/s41598-019-51343-1 PMC6791873

[31] X. Li , J. Li , L. Wang , et al., “The Role of Metformin and Resveratrol in the Prevention of Hypoxia‐Inducible Factor 1α Accumulation and Fibrosis in Hypoxic Adipose Tissue,” British Journal of Pharmacology 173, no. 12 (2016): 2001–2015. 27059094 10.1111/bph.13493 PMC4882491

[32] H. Zhang , H. O. Akman , E. L. P. Smith , J. Zhao , J. E. Murphy‐Ullrich , and O. A. Batuman , “Cellular Response to Hypoxia Involves Signaling via Smad Proteins,” Blood 101, no. 6 (2003): 2253–2260. 12411310 10.1182/blood-2002-02-0629

[33] X. Mingyuan , X. Shengquan , Y. Chenyi , L. Rui , S. Yichen , and X. Jinghong , “Hypoxia‐Inducible Factor‐1α Activates Transforming Growth Factor‐β1/Smad Signaling and Increases Collagen Deposition in Dermal Fibroblasts,” Oncotarget 9, no. 3 (2018): 3188–3197. 29423039 10.18632/oncotarget.23225 PMC5790456

[34] L. Terraneo , E. Virgili , A. Caretti , P. Bianciardi , and M. Samaja , “In Vivo Hyperoxia Induces Hypoxia‐Inducible Factor‐1α Overexpression in LNCaP Tumors Without Affecting the Tumor Growth Rate,” International Journal of Biochemistry & Cell Biology 51 (2014): 65–74. 24704415 10.1016/j.biocel.2014.03.019

[35] S. E. Lacher , D. C. Levings , S. Freeman , and M. Slattery , “Identification of a Functional Antioxidant Response Element at the HIF1A Locus,” Redox Biology 19 (2018): 401–411. 30241031 10.1016/j.redox.2018.08.014 PMC6146589

[36] M. Jaśkiewicz , A. Moszyńska , J. Króliczewski , et al., “The Transition From HIF‐1 to HIF‐2 During Prolonged Hypoxia Results From Reactivation of PHDs and HIF1A mRNA Instability,” Cellular & Molecular Biology Letters 27, no. 1 (2022): 109. 36482296 10.1186/s11658-022-00408-7 PMC9730601

[37] P. Kollmannsberger , C. M. Bidan , J. W. C. Dunlop , P. Fratzl , and V. Vogel , “Tensile Forces Drive a Reversible Fibroblast‐To‐Myofibroblast Transition During Tissue Growth in Engineered Clefts,” Science Advances 4, no. 1 (2018): eaao4881. 29349300 10.1126/sciadv.aao4881 PMC5771696

[38] L. Van De Water , S. Varney , and J. J. Tomasek , “Mechanoregulation of the Myofibroblast in Wound Contraction, Scarring, and Fibrosis: Opportunities for New Therapeutic Intervention,” Advances in Wound Care 2, no. 4 (2013): 122–141. 24527336 10.1089/wound.2012.0393 PMC3656629

