# Supplementary figures and images for: Effects of Oxygen Manipulation on Myofibroblast Phenotypic Transformation in Patients With Radiation‐Induced Fibrosis
Source: Wound Repair Regen. 2025 Aug 18;33(4):e70075. doi: 10.1111/wrr.70075 (PMC12358765; doi:10.1111/wrr.70075)

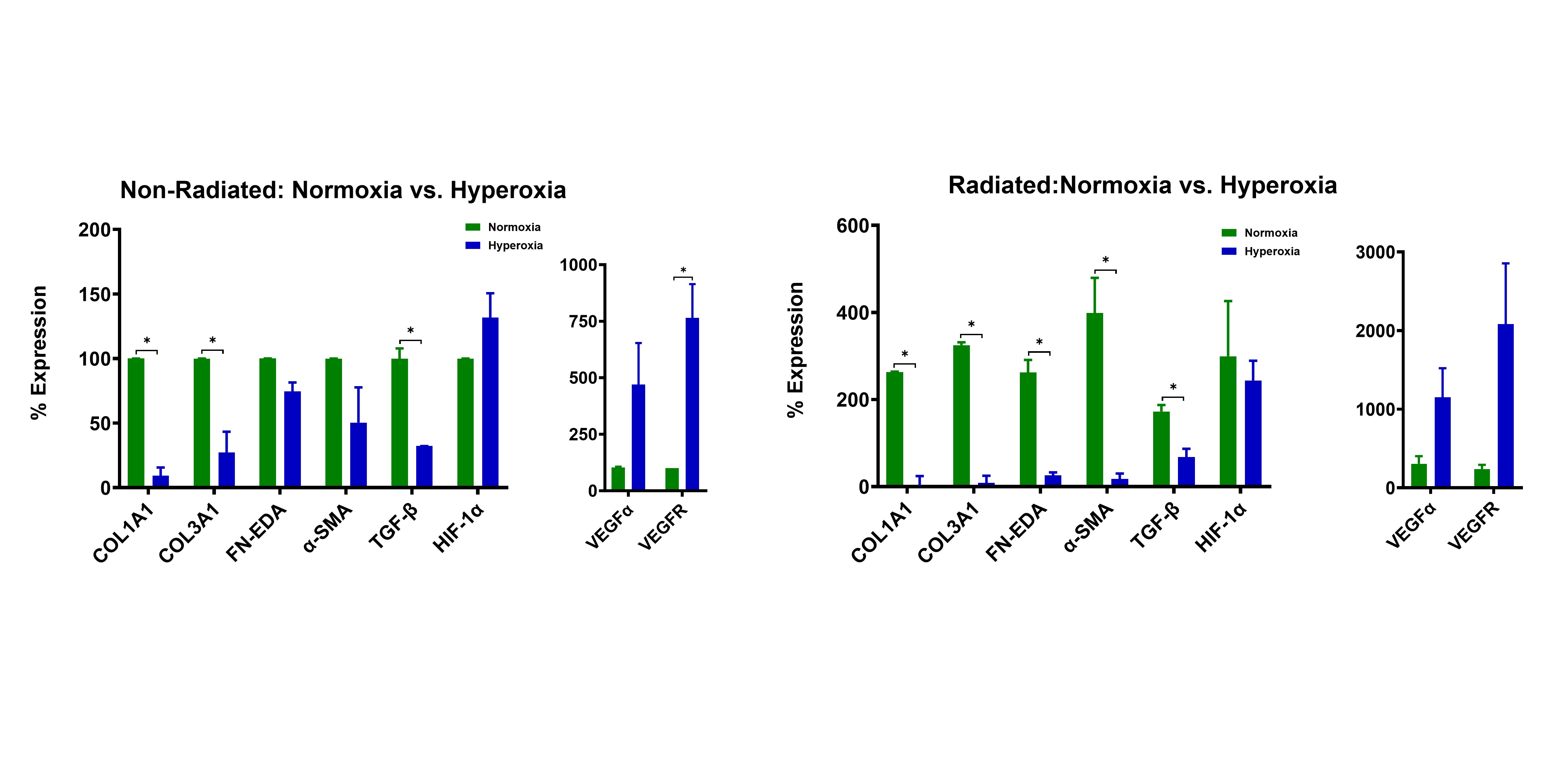

Supplement: Supplementary file 1 — Figure S1: Profibrotic and HIF‐responsive gene expression levels in Normoxia versus Hyperoxia. (A) Non‐radiated: Normoxia versus Hyperoxia. Gene expression levels under normoxic conditions (green) versus hyperoxic conditions (blue) in Non‐radiated tissue. (B) Radiated tissue: Normoxia versus Hyperoxia. Gene expression levels under normoxic conditions (green) versus hyperoxic conditions (blue) in Radiated tissue. [file WRR-33-0-s003.jpg]

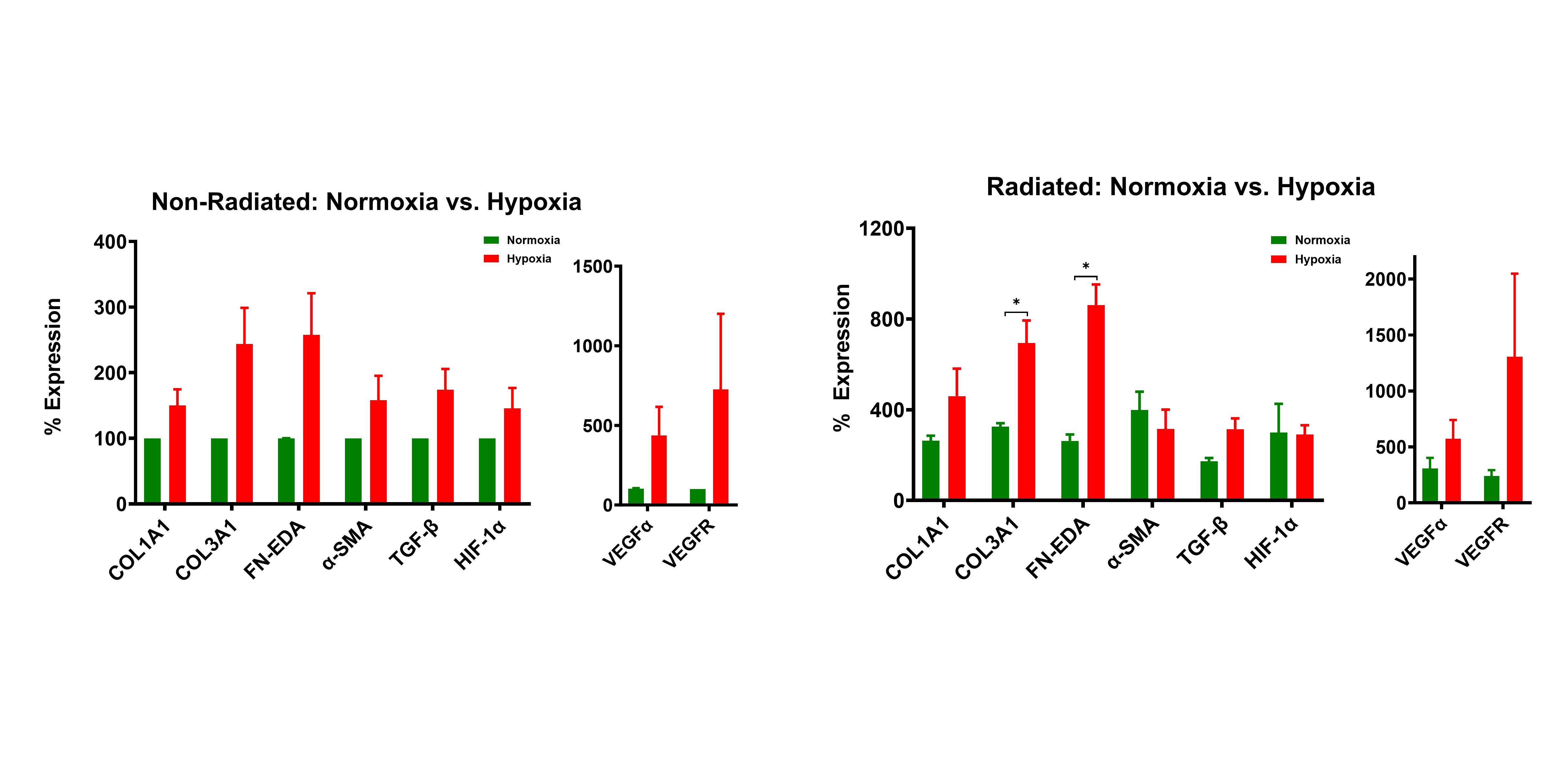

Supplement: Supplementary file 2 — Figure S2: Profibrotic and HIF‐responsive gene expression levels in Normoxia versus Hypoxia. (A) Non‐Radiated Tissue: Normoxia versus Hypoxia. Gene expression levels under normoxic conditions (green) versus hypoxic conditions (red) in Non‐radiated tissue. (B) Radiated Tissue: Normoxia versus Hypoxia: Gene expression levels under normoxic conditions (green) versus hypoxic conditions (red) in Radiated tissue. [file WRR-33-0-s001.jpg]

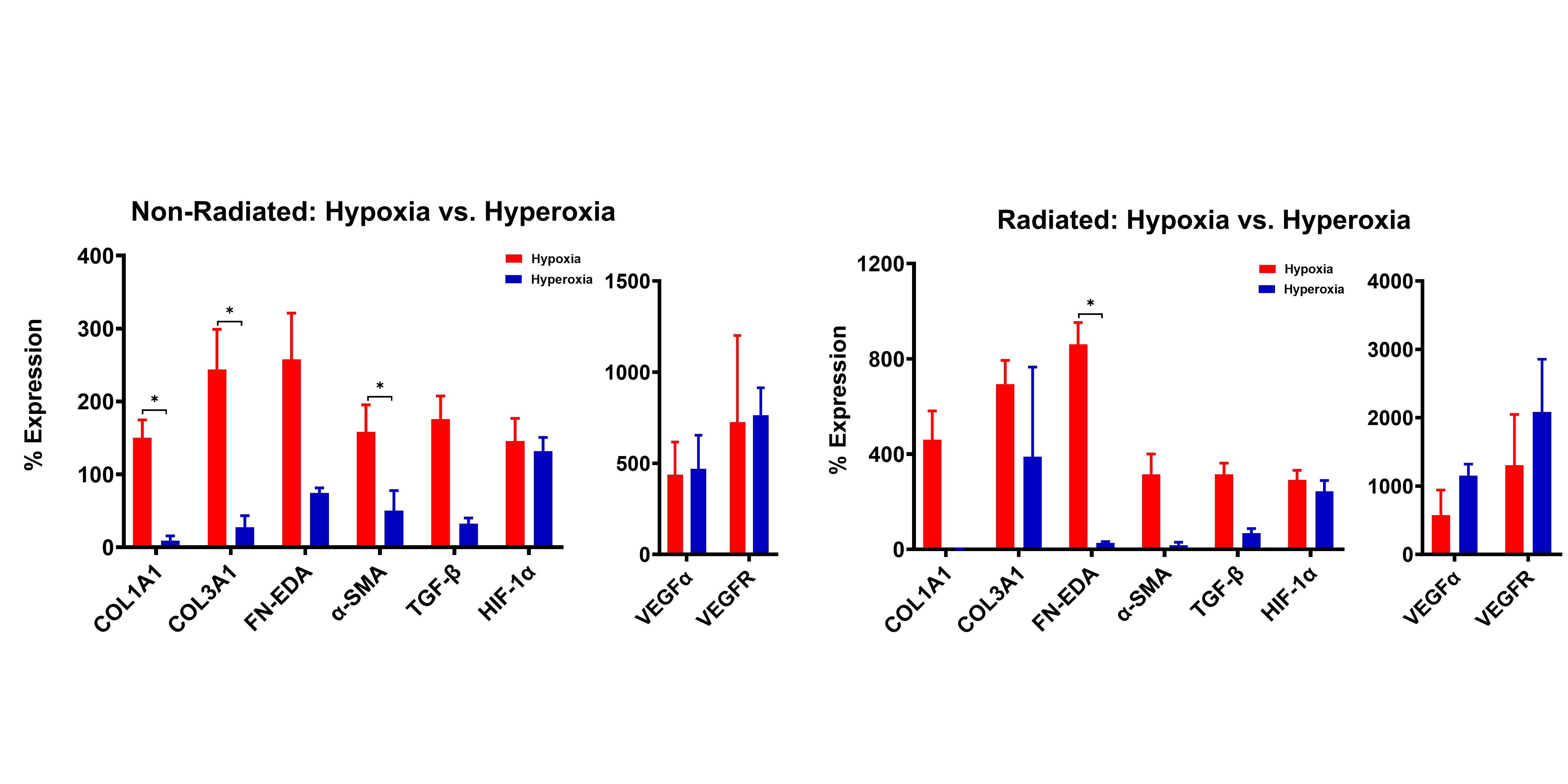

Supplement: Supplementary file 3 — Figure S3: Profibrotic and HIF‐responsive gene expression levels in Hypoxia versus Hyperoxia. (A) Non‐Radiated Tissue: Hypoxia versus Hyperoxia. Gene expression levels under hypoxic conditions (red) versus hyperoxic conditions (blue) in Non‐Radiated tissue and (B) Radiated Tissue: Hypoxia versus Hyperoxia. Gene expression levels under hypoxic conditions (red) versus hyperoxic conditions (blue) in Radiated tissue. [file WRR-33-0-s002.jpg]
